# CXCL1, CXCL10 and CXCL12 Chemokines are Variously Expressed in Acute Myeloid Leukemia Patients Prior and Post Bone Marrow Transplantation

**DOI:** 10.31557/APJCP.2021.22.10.3377

**Published:** 2021-10

**Authors:** Bahar Yazdani, Gholamhossein Hassanshahi, Zahra Mousavi, Zahra Ahmadi, Hussein Khorramdelazad, Alireza Moradabadi, Mohamadreza Shafiepoor, Abbas Fatehi

**Affiliations:** 1 *Department of Hematology and Blood Banking, Kerman University of Medical Sciences, Kerman, Iran. *; 2 *Molecular Medicine Research Center, Rafsanjan University of Medical Sciences, Rafsanjan, Iran. *; 3 *Department of Hematology and Medical Laboratory Sciences, Iranshahr University of Medical Sciences, Iranshahr, Iran. *; 4 *Pistachio safety Research Center, Rafsanjan University of Medical Sciences, Rafsanjan, Iran. *; 5 *Department of Laboratory Sciences, Khomein University of Medical Sciences, Khomein, Iran. *; 6 *Department of Internal Medicine, Faculty of Medicine, Rafsanjan University of Medical Sciences, Rafsanjan, Iran. *; 7 *Department of Pediatrics, Rafsanjan University of Medical Sciences, Rafsanjan, Iran. *

**Keywords:** AML, leukemia, CXC chemokine, CXC chemokine receptor, BMT

## Abstract

**Aim:**

The chemokine-receptor axes play parts in development of leukemia, CXCL1, CXCL10 and CXCL12 are involved in immune responses. Thus, we have examined the serum levels of these chemokines in parallel with their related cognate receptors (CXCR1, CXCR3 and CXCR4) in AML (acute myeloid leukemia) patients prior and post BMT (bone marrow transplantation) therapy.

**Main methods:**

Clinical specimens were collected from 46 AML patients (23 M1 and 23 M3 subtypes) before/after BMT. CXCL1, CXCL10 and CXCL12 concentrations were determined by ELISA. The mRNA levels of the related receptors were detected by QRT_PCR. Data were analyzed by T-test, *χ*^2^ and ANOVA statistical methods in SPSS software version 18. A difference was regarded significant if P value < 0.05.

**Key findings:**

Our results indicated that the elevated levels of CXCL12 in AML patients were remained unchanged after transplantation. The CXCL10 concentration was decreased in patients. All studied chemokines were elevated in BMT patients with history of 9 times PLT transfusion. In patients who received BMT from siblings CXCL1 and CXCL10 have been elevated, whereby they were compared to patients who received BMT from parents while CXCL12 sustained unchanged in groups. Serum measures of CXCL1 and CXCL10 were induced in acute and chronic GVHD patients in compare to these without GVHD.

**Significance:**

According to the results, it can be concluded that these chemokines play fundamental parts in pathogenesis of both AML and BMT. It is worthy to note that chemokines could be used as diagnostic markers alongside with possible promising therapeutic targets.

## Introduction

The malignancy state of AML (acute myeloid leukemia) is characterized as the uncontrolled proliferation of myeloid leukocyte blasts within the bone marrow (BM) with arrested maturation processes. Similarly as, normal precursors, leukemic cells are also able to communicate with their surrounding hematopoietic microenvironment in several paths (Giles et al., 2002). AML is more frequent in elder adults, and is reported to be worsened with increasing age. The incidence of AML increases with age (a median age at its diagnosis is 67 years) (National Cancer Institute. Surveillance, 2009) .

BMT is a promising therapy for acute leukemia which is widely used for therapy of relapsed AML patients. However, the benefits of BMT for patients with late BM relapse or multiple relapses have yet to be firmly clarified (Butturini et al., 1987; Moradabadi et al., 2019) .

The CXC chemokine of stromal cell-derived factor-1 (SDF-1α) which in the latest nomenclature was defined as CXCL12 is described as a potent chemotactic factor for the human CD34 positive pluripotent stem cells (Aiuti et al., 1997). Despite the pro inflammatory chemokines, CXCL12 is constitutively produced and generated by several body organs including human BM (Yazdani et al., 2020; Ponomaryov, 2000). This chemokine plays fundamental parts in homeostatic events including control of leukocyte trafficking, retention of undifferentiated and maturing hematopoietic cells inside BM in both the normal and pathologic states, in addition to CXCR4 and CXCR7, as its related receptors (Leslie RD, 1999;42:3-14; Mousavi et al., 2019) . The disrupted CXCL12/CXCR4 and CXCL12/CXCR7 axes regulate anchorage leading to the release of cells into the circulation(Broxmeyer HE, 2001). It has been well evidenced that degradation of BM originated, CXCL12 by proteolytic enzymes, leading to the release of progenitor and mature cells from the BM towards the periphery in response to granulocyte colony-stimulating factor (G-CSF) -induced mobilization (Petit et al., 2002). 

Investigations revealed that some of the human CXCR4 expressing AML cells are chemo attracted toward a gradient of CXCL12 in a trans endothelial migration assay in vitro (Mohle et al., 1998; Dabiri et al., 2018). 

Since CXCL12 is constitutively expressed by BM stromal cells, it could be speculated that this chemokine contributes to the migration and survival of leukemic blasts during the pathogenesis of acute leukemia (Yoshie et al., 2001). Besides playing a pivotal part in migration, there also exist reports indicating that CXCL12 might be involved in the pathogenesis of leukemia (Nishii et al., 1999). Although, few studies addressed a role for CXCL12 in AML, involvement of CXCL1 and CXCL10 in the pathogenesis of this malignancy yet to be elucidated. Therefore, we designed the current investigation to explore whether if the expression of CXCL1, CXCL10 and CXCL12 in parallel with their cognate receptors is altered before and following BMT in AML patients. Chemokines and their receptors are important in the recruitment of leukocytes to the rejecting allograft. These mediators and their corresponding receptors are specifically expressed in human kidney, heart and lung allografts. Thus, we also aimed to present investigation to detect the CXC chemokines in bone marrow transplanted AML patients and chemokines in relation to clinical status, outcome therapeutic and severity of the disease in BMT received patients.

## Materials and Methods

*Study Subjects*

The research involving human participants and all of the participants had Informed consent. This project was performed during 2015-2017 and 46 AML patients (23 AML- M1 and 23 AML_M3) were enrolled in the study at the Aliebne-Abitaleb Hospital in Rafsanjan, located in South-East of Iran. The type of AML (either M1 or M3) was diagnosed by pathological studies based on the ratio of cell types observed within the BM. 

According to the aim of the study, the serum CXCL1, CXCL10 and CXCL12 levels were examined before and following BMT. We have also explored the expression of CXCR1, CXCR3 and CXCR4 in peripheral blood mononuclear cells (PBMCs) of patients. The occurrence of AML was diagnosed by an expert clinical hemato-oncologist, of course, based on clinical and Para clinical parameters such as BM and peripheral blood smear studies along with chemical staining and also clinical features of the patients. All of the prior BMT samples were collected further extensive chemotherapy.

To collect post BMT samples, patients received entire and complete BMT. All of samples were also obtained from patients who have not shown evidence of either acute, sub-acute or chronic graft rejection. Patients were recruited if exhibited no evidence for infection, injury or inflammation.

Having injury, infection, autoimmunity and known disorder which may change the chemokine expression were considered as excluding criteria. 

All of the clinical samples were collected in the morning following ambulatory visits and 5 mL of blood was taken from the patient. Serum samples were kept at -20°C for further analysis in the Molecular Medicine Research Center, Rafsanjan University of Medical Sciences, Rafsanjan-Iran.

We selected healthy individuals from the Kerman population as the control group and were then matched with AML patients about demography, including age and sex status.

All of the participants have filled a written consent out form and the study program was approved by the Rafsanjan University of Medical Science regional ethical committee.

*Assessment of chemokines by ELISA*

The CXCL1, CXCL10 and CXCL12 serum levels were measured by ELISA (R&D systems, UK). All of assays were conducted according to manufacturer’s guidelines.

*RNA isolation and QRT-PCR*

The PBMCs were harvested using density gradient separation (Ficoll–Hypaque; NyCoMed, Oslo, Norway; specific density 1.077) from the Peripheral blood samples of patients and then, the total RNA was isolated from PBMCs employing trizol in accordance with the manufacturer’s instructions (Invitrogen, USA). cDNA was synthesized from total RNA (Thermo Scientific, USA) all of assays were conducted according to manufacturer’s guidelines. The reference gene of GAPDH was selected based on a uniform expression in all samples. QR-PCR was performed for mRNA detection of chemokine receptors CXCR1, CXCR3, CXCR4, by applying Power SYBR Green PCR Master Mix (Amplicone) in a Rotor-Gene Q system the following conditions: 95°C for 15 min and 40 cycles at 95°C for 15 s and 60°C for 1 min. The specific Primer sequences for chemokines receptors are listed in Table 1. 

*Statistical analysis*

Statistical analysis of the data was assessed by* χ*^2^, T-Test and ANOVA using SPSS software version 18 with power test of 90%. The difference regarded significant, if the P value was less than 0.05.

## Results

*Patients*

To investigate the fundamental role played by the CXC chemokines, including CXCL1, CXCL10 and CXCL12 in AML patients prior and post BMT, the present study was carried out on 46 AML patients (23 AML-M1 and 23 AML-M3). 

Our results showed that the mean age in male patients was 24(64.86%). Our findings also demonstrated that the mean age in female patients was 13(35.1%). 5(3.1%) of patients had familial history of cancer (Table 2).

*Level of Serum Chemokines*

Further data analysis, our data indicated that the expression of CXCL1, CXCL10 and CXCL12 was not associated with the gender of AML patients. We did not also find a relationship between the age and expression of chemokines in AML patients. We did find a significant difference in the CXCL1 level between M1 subtype and M3 subtype (P<0.01) (Table 3). We have observed a remarkable difference between AML subtypes regarding CXCL10 expression, M1 and M3 subtypes (P<0.01) (Table 3). A marked difference was also found between CXCL12 levels in patients with M1 subtype and M3 subtype (P<0.01) (Table 3).

**Figure 1:**
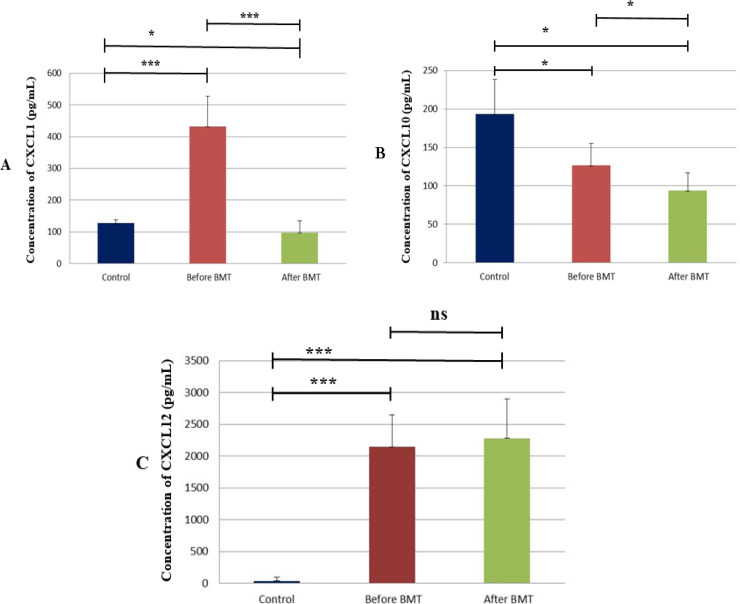
The Serum Concentration of Chemokines before and after BMT in AML Patients with the Control Group. The results of ELISA have been shown. (A) There was a significant difference between the control group and before BMT (*** p < 0.001). The serum concentration of CXCL1 was significantly decreased after BMT compared with before BMT (*** p < 0.001). There was a significant difference between the control group and after BMT (* p < 0.05). (B) There was a significant difference between the control group and before BMT (* p < 0.05). The serum concentration of CXCL10 was significantly decreased after BMT compared with before BMT (* p < 0.05). There was a significant difference between the control group and after BMT (* p < 0.05). (C) There was a significant difference between the control group and before BMT also, the control group and after BMT (*** p < 0.001). The serum concentration of CXCL12 was not differed significantly before and after BMT (p ˃0.05)

Our findings demonstrated that the circulating concentration level of CXCL1 was significantly decreased in both AML subtypes after BMT (P<0.001). Also, there was a significant difference between the control group and before BMT (p < 0.001). There was a significant difference between the control group and after BMT (p < 0.05) (Figure 1A).

Current results indicated that the serum concentrations of CXCL10 were considerably attenuated in both AML subtypes after BMT (P<0.05). There was a significant difference between the control group and before BMT (p < 0.05). The serum concentration of CXCL10 was significantly decreased after BMT compared with before BMT (p < 0.05). There was a significant difference between the control group and after BMT (p < 0.05). (Figure 1B). 

Our findings have also indicated that, although, the CXCL12 circulating concentrations were slightly increased in both subtypes of AML patients before and after BMT but it was not significantly differed prior and post BMT. There was a significant difference between the control group and before BMT also, the control group and after BMT (p < 0.001). The serum concentration of CXCL12 was not differed significantly before and after BMT (p ˃0.05) (Figure 1C).

These results also showed that the CXCL1 concentration in patients who have received platelets three, six and nine times was 56.32±14.96 pg/mL, 65.98±10.91 pg/mL and 88.45±5.58 pg/mL, respectively.

Our findings also demonstrated an increased level of CXCL10 in BMT transplanted AML patients with a history of receiving platelet nine times before BMT in comparison to three and six times groups. The mean concentration of CXCL10 in three, six and nine times platelet transfusion was 43.09±9.05 pg/mL, 52.41±9.59 pg/mL and 83.78±10.25 pg/mL, respectively. Our data analysis indicated a remarkable difference between patients who have received platelet transfusion three and six times with nine times (P<0.05) while CXCL10 was not significantly differed between patients who were transfused for three and six times. 

Our results showed that the concentration of CXCL12 in BMT transplanted AML patients having a history of three, six and nine times of platelet transfusion, (before BMT) was, 1231±287 pg/mL, 1463.03±309.7 pg/mL and 2249.16±191.02 pg/ml, respectively. A considerable difference has been observed between three and six times with nine times platelet transfusion (P<0.04) while a significant difference was not observed between AML patients received platelets for three and six times.

Our data showed that, however all of examined chemokines were elevated in patients who had a history of packed cell transfusion, but this difference was not considerably significant (Table 4).

These results demonstrated a significant difference between siblings received BMT and patients who received their BMT from their parents regarding CXCL10 and CXCL1. Regarding, the mean concentration of CXCL10 was 84.2±7.5 pg/mL and 52.8±4.3 pg/mL in sibling and parents group, respectively. The mean concentration of CXCL1 was 87.5±4.3 pg/mL and 46.5±5.2 pg/mL in sibling and parents group, respectively (P<0.05). A significant difference between the level of CXCL12 in sibling and parents received AML patients was not observed (Table 4). Our studied sex matched patients had lesser CXCL10 and CXCL1 in comparison to mismatch patients regardless of the type of male/female or female/male BMT donors. The mean concentration of CXCL10 in male/female, female/male and matched BMT received AML patients was 85±6.3 pg/mL, 82±4.7 pg/mL and 44.3±3.7 pg/mL, respectively. Significant difference was observed between matched and male/female, female/male BMT received AML patients. (P<0.05). The mean CXCL1 in mismatched and matched BMT received AML patients was 87±8.5 pg/mL, 79±12.2 pg/mL and 54.3±7.6 pg/mL, respectively. A significant difference was observed between matched and mismatched male/female and female/male, BMT received patients (P<0.05).

*Expression of receptors*

**Figure 2:**
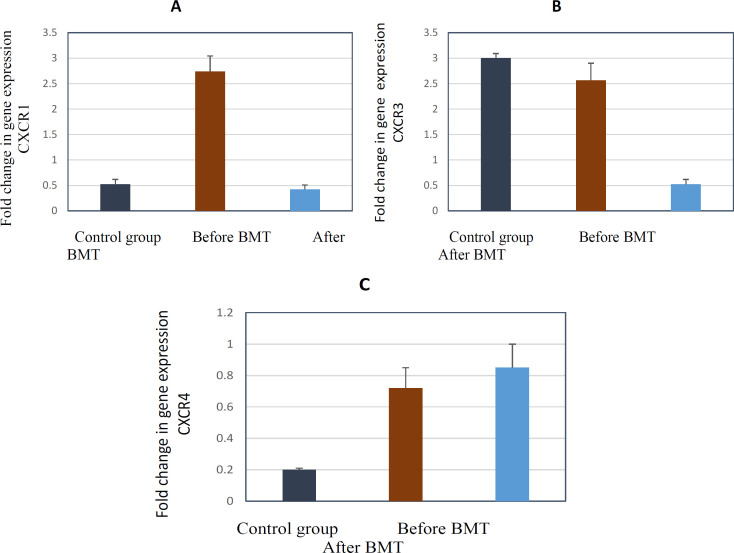
The Gene Expression of Receptors before and after BMT in AML Patients Alongside with Control Group. The result of Sybr-green real-time PCR using specific primers has been shown. All values were normalized to GAPDH. (A) The gene expression of CXCR1 was significantly decreased after BMT compared with before BMT (P < 0.001). (B) The gene expression of CXCR3 was significantly decreased after BMT compared with before BMT (P < 0.05). (C) The gene expression of CXCR4 was not differed significantly before and after BMT (P ˃0.05)

Our QRT_PCR analysis revealed that the gene expression of CXCR1 and CXR3 was significantly decreased after BMT (P < 0.001, P < 0.05 respectively), but the gene expression of CXCR4 was not differed significantly before and after BMT (P ˃0.05) (Figure 2).

**Table 1:** Primers Used for Real-Time RT-PCR

Gene	Forward primer (5´- 3´)	Reverse primer (5´- 3´)
CXCR1 receptor	GCAGCTCCTACTGTTGGACA	GGGCATAGGCGATGATCACA
CXCR3 receptor	TACCTTGAGGTTAGTGAACGTCA	CGCTCTCGTTTTCCCCATAATC
CXCR4 receptor	AATGGGCTCAGGGGACTATG	CTGTACTTGTCCGTCATGCT
GAPDH	GAAGGTGAAGGTCGGAGTC	GAAGATGGTGATGGGATTTC

**Table 2:** Indicates Demographic Characteristics of BMT Patients

Variable	BMT patients
Age (year)	
Male, year (%)	24(64.86%)
Range	(3-61 years)
Gender	
Female, year (%)	13(35.1%)
Range	(4-39 years)
Familial history	5(3.1%)

**Table 3:** Indicated the Expression of CXCL1, CXCL10 and CXCL12 before and after BMT in AML Patients based on FAB Classification

	Chemokine serum level * Mean ± SEM AML			
	M1**		M3	
	Before	After	Before	After
CXCL1	281.3±30	58.3±6.3	312.4±27.5	83.1±7.4
CXCL10	55.23±4.6	50.58±8.3	126.74±37.9	98.5±22.3
CXCL12	1386.25±82.31	1588.26±58	1986.75±389.3	2114.59±363.86

*, pg/mL as measured by ELISA; **, Significant difference between M1 and M3.

**Table 4:** Demonstrates Some Clinical Aspect of Patients Relative to Chemokine Expression

Clinical aspects of BMT patients	CXCL12	CXCL10	CXCL1
Platelet transfusion before BMT			
Three times 16 (43.24%)	a123±287	a43.09±9.05	a56.32±14.96
Six times 10 (27.02%)	a1463±309.7	a52.41±9.59	a65.98±10.91
Nine times 11 (29.7%)	2294.16±191.02	83.78±10.25	88.45±5.58
Packed cell transfusion before BMT			
Three times 21 (56.15%)	1298±181.9	41±5.58	53.35±12.69
Six times 6 (16.2%)	1302±217	50.31±9	54.45±8.29
Nine times 10 (27.02%)	1440±153.6	60±7.1	64.39±9.8
Donor Status			
Siblings 31(83.78%)	1521.4±210.7	b84.2±7.5	b 87.5±4.3
Parents 6 (16.26%)	1480.6±173.2	52.8±4.3	46.5±5.2
Sex mismatched			
Male donor/Female recipient 7(18.9%)	1972±312	c 85±6.3	c 87±8.5
Female donor/ Male recipient 9 (2.32%)	1843±412	c 82±4.7	c 79±12.5
Sex matched 21 (56.15%)	2217±215	44.3±3.7	54.3±7.6
GVHD			
Acute 10 (27.02%)	2232.3±292	d 87.3±5.2	d 91±5.4
Chronic 15 (40.54%)	1963±182	d67.3±4.7	d59.6±6.2
Without 12(32.43%)	2113.4±147.6	46.2±5.3	43.6±8.2
Number of dead due to GVHD7 (18.9%)	NA	NA	NA

a, Significant difference with nine times platelet transfusion; b, Significant difference with parents received BMT group; c, Significant difference with sex matched BMT; d, Significant difference without GVHD group.

## Discussion

We have analyzed some CXC chemokines in AML patients (AML-M1 and M3), before and after BMT. We have also examined the expression of specific receptors for these chemokines (CXCR1 for CXCL1, CXCR3 for CXCL10 and CXCR4 for CXCL12), employing Real time-PCR technique. These results showed that the circulating levels of CXCL12 were considerably elevated in AML patients (both type) before and after BMT by ELISA.

In addition to malignant AML cells, CXCL12 is also shown to be constitutively produced by immature osteoblasts, stromal and endothelial cells which present in BM tissue (Wright et al., 2002). In agreement with our study, investigators reported that the CXCL12 level was increased in BM following irradiation and chemotherapy (Peled et al., 2000). CXCL12 involved in the first step of HSCs transplantation. AMD3100 is a special antagonist to disentangle HSCs from stromal cells. In one study, scientists showed one approach of HSCT that delivering allogeneic endosteal BMCs (bone marrow cells) into recipient BMs. They indicated that this method could facilitate the homing efficiency of HSCs allogeneic transplantation. This advantage resulted from the expression of CXCL-12. This method provides new insights into finding new HSCs sources. The endosteal BMCs could be prepared a source of donor HSCs and the intra-bone approach offers an efficient transplantation to induce donor hematopoietic and stromal cell reconstitution (Inoue et al., 2006; Fekri-SoofiAbadi et al., 2019). 

To the best of our knowledge this study is the first addressing expression of CXCL1, CXCL10 and CXCL12 and their receptors in BMT received AML patients, however, it has been shown that most often AML cells constitutively produce the chemotactic chemokines such as CXCL10 and CCL5. These results showed that there was a closed relationship between the differentiation (based on FAB classification of AML) and chemokine expression. So that more differentiated cell types secreted more chemokines (Table 3).

Animal studies indicated that processes of homing and engraftment of human stem cells in immune-deficient mice is dependent on the CXCR4 expression and the BM production of CXCL12, which functioning in the survival of human and murine stem cells. However, the role of CXCL12/CXCR4 axis on control of migration of AML cells as well as disease progression yet to be well understood. More recent investigations demonstrated that the neutralization of CXCR4 could be considered as a potential for therapy of AML 15-(Tavor et al., 2004) .

Studies by Sarris et al., (1996) indicated that supplementation of cultured AML cells with recombinant CXCL10 can inhibit their proliferation. Taken together with our results, also CXCL10 could probably be used as an anti-leukemic agent in human AML patients (Hassanshahi et al., 2008) . CXCR3 play a role in the activation of NK cells, T cells and monocytes. Therefore, some studies indicated that CXCL10 was elevated at cGVHD in some grafts (Kariminia et al., 2016). Therefore, we decided to evaluate CXCL10 in AML patients after BMT. We observed that CXCL10 and CXCL1 in patients with GVHD was considerably higher than the patients without GVHD (P < 0.0001) while the serum level of CXCL12 was similar in both patients with GVHD and without GVHD (P > 0.05). B Lamarthe and colleagues inhibited CXCR3 and showed that the severity of GVHD is reduced in mice. Thus, these investigations recommended that CXCL10 signaling pathway is essential in human GVHD (Lamarthée et al., 2016). Along with this study and our study, it can be concluded that inhibition of the CXCR3/CXCL10 signaling can reduce GVHD in AML patients after BMT.

CXCL1 was markedly decreased following BMT and this reduced level is perhaps due to decreased cellular sources of this chemokine because this chemokine is produced by several BM resident and migratory cell types (Abdolmaleki and Sohrabi, 2018). One of the possible mechanisms which may explain the decreased levels of both CXCL1 and CXCL10 following BMT, is their nature as inducible chemokine. Most often inducible chemokines, are expressed by following either inflammatory responses insult or injury states and/or infection (Abdolmaleki and Sohrabi, 2018). Hence, it is expected that BM transplanted AML patients, received extensive treatment for the control of inflammatory and infection and maybe pathogen free. Therefore, maybe due to the lack of inflammation and infection circulatory levels of these inducible chemokines are decreased after BMT. Data analysis showed that the expression of CXCR1 and CXCR3 decreased post BMT and the expression of CXCR4 did not change which is in line with changes in chemokines.

Furthermore, regulatory consensus elements for heat shock, NF-KB (Ohmori et al., 1995) transcription factors are present in both CXCL1 and CXCL10 genes (Gholamhossein Hassanshahi, 2009; 7 (1): 1-9. 28) and their down-regulation is probably due to the absence of these stimulatory situations following BMT, as a result of upstream signals (Akpınar et al., 2013). Additionally, based on the role of CXCL10 on inhibition of neovascularization may indirectly decrease the complications of leukemia and BMT in the patient, leading to a better prognosis. Therefore, as a future work the designing of leukemia animal models with employing recombinant CXC chemokines CXCL1, CXCL10 and CXCL12 is proposed for the examination of the role and ability of these chemokines in control of AML complications. It is worthy to note the CXC chemokines properties as therapeutic agents following BMT and graft survival in the leukemia and other BMT received patients as well as animal models.

In the current study we also demonstrated that patients who received either 3 and/or 6 times platelet (PLT) transfusion had lesser measures of all studied chemokines in comparison to, 9 times platelet transfusion (Table 4). Because in our country HLA- antigens are not routinely controlled before PLT-transfusion, this probably could be explained by the fact that maybe HLA mismatched platelet transfusion before BMT could stimulate immune system and also cellular sources of these chemokines. This could be examined in future studies. Having a history of packed cell transfusion before BMT did not altered the level of none of our examined CXC chemokine (Table 4). However, we observed a minor change in chemokines circulating level but it was not considerable, this also could possibly be due to the minor antigenicity of RBC and deposits of plasma proteins present in transfused blood unites and also transfusion-related complications(Nydegger et al., 2005). Although, CXCL12 circulating level was not affected by the type of donor and BMT patients who received bone marrow from siblings or parents showed similar pattern of CXCL12 levels, but inducible and inflammatory chemokines (CXCL1 and CXCL10) were elevated in BMT patients who received bone marrow from their siblings. This could probably be due to: a) Sex differences probably affect the chemokine level as we also shown that sex mismatch BMT patients expressed more inducible CXC chemokines in comparison to sex matched donor/recipients (Table 4). b) These discrepant data could probably be due to our small sample size and we need to extend our studies with a wider sample size in different groups. c) This could also be due to age differences because almost sibling donors were in a similar age range with patients while parents were elder than BMT patients.

In conclusion, all studied chemokines were elevated in BMT patients with history of 9 times PLT transfusion. Therefore, this probably could be explained by the fact that maybe HLA mismatched platelet transfusion before BMT could stimulate immune system and also cellular sources of these chemokines. In patients who received BMT from siblings CXCL1 and CXCL10 have been elevated, whereby they were compared to patients who received BMT from parents while CXCL12 sustained unchanged in groups. Serum measures of CXCL1 and CXCL10 were induced in acute and chronic GVHD patients in compare to these without GVHD. So, inhibiting of CXCL1 and CXCL10 can aimed the good prognostic for BMT. In conclusion, taken together in an overall view, our findings in harmony with previous studies suggest that these chemokines maybe play fundamental parts in the pathogenesis of both AML and BMT. It is worthy to note that chemokines may be used as diagnostic markers alongside possible promising therapeutic targets in future.

## Author Contribution Statement

GH.H. proposed the original concept and designed the experiment and supervised all aspects of the work. B.Y., Z.M., A.M., H.KH. Z.A., M.SH. A.F., and A.S. equally participated in the data acquisition and analysis. All authors contributed to writing the manuscript. GH.H. provided critical reviews in order to promote the manuscript.
